# Research on the allocation mechanism of grassland ecological compensation among different industries based on ecosystem product value accounting

**DOI:** 10.1371/journal.pone.0349316

**Published:** 2026-05-15

**Authors:** Li Ma, Yaojie Wu

**Affiliations:** 1 College of Economics, Lanzhou University of Finance and Economics, Gansu, China; 2 Academy of Animal Husbandry and Veterinary Sciences, Qinghai University, Qinghai, China; Sophia University: Jochi Daigaku, JAPAN

## Abstract

The manifestation and allocation of the positive externalities of grassland ecosystem products are critical to realizing the value of ecological compensation. Previous studies on ecological compensation have largely overlooked the heterogeneity of ecosystem products within regions and the differences in compensation among various industrial sectors, particularly the alignment and regulatory role between industrial structure and ecological compensation. In response, this study shifts the focus from interregional horizontal compensation to intraregional structural compensation, emphasizing the rational allocation mechanism of ecosystem product value among different industrial actors. Based on meteorological monitoring data and land-use data, the value of grassland ecosystem products is estimated using the water balance method and soil erosion equations. Furthermore, an inter-industry ecological compensation allocation model is constructed based on the consumption value of grassland ecosystem products and the proportion of industrial structure. The results show that: (1) From 2013 to 2023, none of the four major pastoral regions are required to bear ecological compensation for carbon sequestration and oxygen release consumed by the livestock industry; however, the average ecological compensation surplus decreased from 7.38 × 10⁸ CNY to 7.09 × 10⁸ CNY. (2) Most regions are required to bear ecological compensation for air purification consumed by the industrial sector, while some regions in Inner Mongolia and Xinjiang are also required to compensate for water conservation consumed by industry. (3) No region is required to bear ecological compensation for water conservation and soil retention consumed by the tourism industry; however, regions such as Turpan and Alxa are required to compensate for carbon sequestration and oxygen release consumed by tourism, with the average compensation increasing from 1.35 × 10⁶ CNY to 1.54 × 10⁷ CNY.

## Introduction

Grassland is the second-largest terrestrial ecosystem in China, predominantly distributed across the country’s four major pastoral regions. Its total area reaches 2.645 × 10⁸ hm², accounting for approximately 70% of the national grassland area, and it provides a wide range of ecosystem products, including provisioning, regulating and supporting, and cultural services. The realization of ecosystem product value constitutes a crucial pathway for transforming “lucid waters and lush mountains” into “invaluable assets” [1]. Grassland ecosystem products are an essential component of the national green ecological barrier and ecological security, as well as a fundamental basis for ensuring food security and promoting traditional grassland culture. The value of grassland ecosystem products reflects the substantial ecological and economic benefits embedded within grasslands and plays a vital role in reconciling ecological conservation with economic development. However, due to the public goods nature of ecosystem products, the behaviors of grassland ecosystem product providers generate positive externalities, leading to the incomplete realization of their value [2]. Practical experience both domestically and internationally indicates that achieving ecosystem product value requires the establishment of a government-led, market-oriented compensation mechanism for quasi-public ecological goods [3].

Ecological compensation is a mechanism through which beneficiaries of ecological protection provide financial resources, technical support, or policy incentives to contributors of ecological conservation [4]. It serves as a key approach for enhancing and realizing ecosystem product value and constitutes an important instrument for ensuring the sustainable development of ecosystems [5]. Through economic means, ecological compensation aims to restore ecosystems by offsetting the losses and degradation of ecosystem services caused by economic activities [6], thereby contributing significantly to environmental, social, and economic sustainability. Well-designed ecological compensation mechanisms not only promote ecosystem health but also generate sustainable economic benefits [7].

Current approaches to ecological compensation accounting mainly include three categories. First, from the perspective of ecological protection costs, methods such as the direct cost approach and opportunity cost approach are employed to estimate the costs incurred in maintaining ecosystem services [8]. These methods, grounded in actual expenditures and forgone benefits, emphasize compensation for “inputs and sacrifices,” featuring clear property-right implications and institutional applicability. However, they primarily compensate for the costs borne by protectors and fail to fully capture the true supply and external benefits of ecosystem services. Second, from the perspective of ecosystem service functions and values, ecological compensation is estimated based on the functions and monetary value of ecosystem services, using methods such as the ecosystem service value approach [9], contingent valuation method [10], and carbon trading and carbon balance approaches [11]. These methods are rooted in ecosystem service theory and aim to internalize externalities by monetizing ecological functions, thereby emphasizing the marginal contribution of ecosystems to human well-being. Third, from the perspective of energy and emergy transformation within ecosystems, ecosystem supply and demand are converted into standardized energy or solar emergy units to serve as the basis for compensation, with representative methods including the ecological surplus method and the emergy ecological footprint approach [12]. These methods, based on principles of energy conservation and system metabolism, unify different ecosystem products and services into a standardized energy metric, enabling the analysis of supply–demand structures and operational mechanisms at the material–energy flow level. However, their interdisciplinary complexity and high requirements for data completeness and model construction limit their application in macro-level decision-making.

In comparison, value-oriented approaches, centered on ecosystem service functions and their monetary valuation, achieve an effective integration of ecological processes and economic assessment. Their advantages lie not only in overcoming the limitations of cost-based approaches—by shifting the basis of compensation from inputs to ecosystem service outputs and their contributions to social welfare—but also in enhancing comparability and communicability across ecological functions, spatial units, and stakeholders through a unified monetary metric. This facilitates cross-regional negotiation, benefit balancing, and policy implementation.

As an important institutional tool for coordinating ecological protection and regional development, ecological compensation has gradually formed a research framework centered on vertical and horizontal compensation [13]. Vertical ecological compensation is primarily reflected in fiscal transfer payments from the central government to local governments. Its theoretical foundation lies in public goods provision and the internalization of ecological externalities, emphasizing top-down institutional arrangements to offset opportunity costs in ecologically protected regions [14]. Existing studies mainly focus on compensation standard accounting [15], fund allocation efficiency [16], and policy performance evaluation [17], indicating that while vertical compensation provides stability and institutional guarantees for safeguarding the interests of ecological function areas, it also suffers from insufficient incentives and delayed responses to regional heterogeneity [18]. In contrast, horizontal ecological compensation is constructed based on the supply–demand relationship of ecosystem services among regions, typically within river basins or cross-regional ecosystems. It achieves direct compensation among stakeholders through market-based or negotiated mechanisms, with theoretical foundations closer to the Coase theorem and ecosystem service value transfer [19]. Existing studies demonstrate that horizontal compensation has significant advantages in enhancing incentive compatibility and improving resource allocation efficiency. For instance, Lei et al. introduced environmental reward and penalty policies into horizontal fiscal mechanisms, breaking through traditional vertical transfer payment models and achieving both incentives and constraints for ecological-economic development [20]. Zhang et al. proposed a resource allocation compensation framework based on the concept of “green water credit,” effectively coordinating upstream and downstream development and improving resource allocation efficiency [21]. However, the implementation of horizontal ecological compensation relies on clear property rights and well-established negotiation mechanisms, resulting in relatively high institutional costs [22].

Overall, vertical and horizontal ecological compensation exhibit complementary governance logics and implementation pathways: the former emphasizes institutional guarantees and equity, while the latter focuses on efficiency improvement and incentive optimization. Together, they constitute the developmental direction of a multi-level ecological compensation system. Nevertheless, existing research on ecological compensation mechanisms predominantly takes a single ecosystem as the analytical starting point and focuses on interregional horizontal compensation, forming an analytical paradigm centered on interregional benefit regulation. To some extent, this approach overlooks the structural differences in ecosystem service supply and demand within regions, particularly failing to adequately identify the heterogeneity in compensation responsibilities and benefit distribution among different types of ecosystem products and their corresponding industries. Moreover, limited attention has been paid to the coupling relationship between ecological compensation mechanisms and industrial structures from the perspectives of industrial optimization and resource allocation efficiency, resulting in a mismatch between compensation standards and industrial development patterns, thereby affecting both the equity and effectiveness of ecological compensation.

Therefore, this study extends the analytical perspective from interregional horizontal compensation to intraregional structural compensation, focusing on the rational allocation mechanism of ecosystem product value among different industrial actors. Based on meteorological monitoring data, land-use data, and net primary productivity (NPP) data, this study estimates the value of grassland ecosystem products using methods such as the water balance approach, soil erosion equations, and atmospheric pollution models. Furthermore, an inter-industry ecological compensation allocation model is constructed based on the consumption value of grassland ecosystem products and the proportion of industrial structure. The study aims to establish a “government-led and industry-participatory” ecological compensation mechanism, reduce the practical constraints imposed by regional fiscal pressure, and provide a theoretical basis for balancing regional industrial development with ecological compensation and optimizing intraregional ecological compensation systems.

## 1. Research area

**Fig 1:**
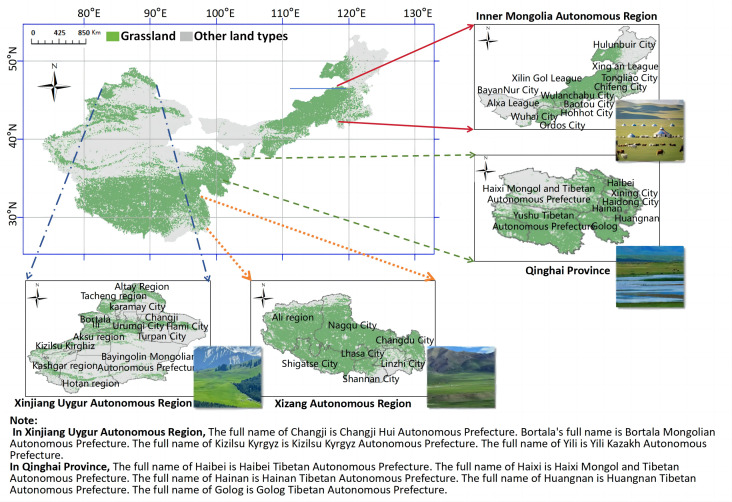
Research area. Map quoted from the National Geospatial Information Research Directory Service System - www.webmap.cn. Map Approval Number GS(2024)0650.

This study selects 41 prefecture-level cities and regions from the four major pastoral areas of Inner Mongolia, Xinjiang, Qinghai, and Tibet, based on principles such as regional grassland type characteristics, grassland area proportion, regional industry characteristics, and evenly distributed sample points (Fig 1). The total grassland area of the selected samples accounts for 60% of the total grassland area in China, and the total output value of the grass industry in these samples represents more than 90% of the total output value of the grass industry in China. The types of grasslands covered include various types such as thermal shrub-grassland, warm grassland, temperate grassland, alpine meadow, lowland meadow, and mountain meadow, making the sample highly representative.

## 2. Research methods and data sources

### 2.1 Grassland ecological product value estimation model

This study’s accounting method for the value of ecological products follows the “Technical Guidelines for the Accounting of Gross Ecosystem Product (GEP) of Terrestrial Ecosystems” and primarily calculates two main categories: direct products and indirect products. Among the indirect products, regulatory services include four subcategories: water conservation, soil retention, carbon sequestration and oxygen release, and air purification, as shown in Table 1 [23].

**Table 1:** Valuation method for grassland ecological products.

Product Categories	Product type	Code	Value estimation method	Accounting method
**Direct Products**	livestock products	V_A_	Statistical yearbook	Market Value Method
**Indirect Products**	water conservation	V_B1_	Shadow Reservoir Cost Method	replacement cost
	Soil Conservation	V_B2_	Soil erosion equation	replacement cost
	carbon fixation and oxygen release	V_B3_	Net Ecosystem Productivity Law	Market Value Method
	Air Purification	V_B4_	Air pollution model	Market Value Method

(1) Livestock Products

This study adopts the existing actual carrying capacity indicator to reflect the maximum supply capacity of livestock products by the grassland ecosystem. The calculation formula is as follows:


$$\begin{array}{c} \text{SC}=\frac{\text{A}+\text{F}}{\text{H}\times \text{SSE}} \end{array}$$ (1)

$$\begin{array}{c} V_{A}=SC\times P_{A} \end{array}$$ (2)

In the equation, SC denotes the actual livestock carrying capacity; A represents the product of aboveground edible forage yield per unit area and the actual grassland area; F denotes forage input; H represents the actual grazing days; SSE denotes the forage requirement per standard sheep unit, with a value of 1.8 kg [24]; V_A_ represents the direct product value of grassland; and P_A_ denotes the unit price of sheep in the given year.

(2) Water Conservation

The water balance method is employed to estimate water conservation capacity [25]. This method considers only the water inputs and outputs of the ecosystem to estimate the water conservation volume and subsequently derive its economic value. The formula is as follows:


$$\begin{array}{c} V_{B1}=Wca\times Src\times S \end{array}$$ (3)

$$\begin{array}{c} Wca=R-Q-Run \end{array}$$ (4)

$$\begin{array}{c} Run=\alpha \;\times \;R \end{array}$$ (5)

In the equation, V_B_₁ represents the value of water conservation; Src denotes the average construction cost of reservoir storage capacity in the given year; S represents the grassland area; Wca denotes the water conservation volume; R is precipitation; Q represents actual evapotranspiration; Run denotes surface runoff; and α is the runoff coefficient for different landscape types. In this study, grassland types mainly include alpine steppe (α = 6.45%) [25], alpine meadow (α = 8.2%) [25], temperate steppe (α = 3.94%) [25], and temperate meadow steppe (α = 9.13%) [25].

(3) Soil Conservation

The soil conservation function of grassland ecosystem products primarily reflects the reduction of land degradation [26]. This study estimates soil conservation value using the soil erosion equation and the replacement cost method. The formula is as follows:


$$\begin{array}{c} V_{B2}=\frac{P\times T}{\rho \times H} \end{array}$$ (6)

$$\begin{array}{c} T=S\times (Ap\;-\;Ar) \end{array}$$ (7)

In the equation, V_B_₂ represents the value of soil conservation; P denotes the average annual return of grassland; T represents the total amount of soil conserved; ρ is soil bulk density; H denotes the soil depth considered; S represents the grassland area; Ap is the potential soil erosion modulus; and Ar is the actual soil erosion modulus. According to existing studies, the potential erosion modulus is 319.8 t·(hm²·a)⁻¹ [27], the average erosion modulus is 0.93 m³·(hm²·a)⁻¹ [27], soil bulk density is 1.35 t·m ⁻ ³ [26], and the calculation depth of grassland soil is taken as an average thickness of 0.5 m [26].

(4) Carbon Sequestration and Oxygen Release

In this study, carbon sequestration and oxygen release refer to the amounts of CO₂ fixed and O₂ released. Vegetation absorbs atmospheric CO₂ and releases O₂ through photosynthesis. Thus, the quantities of CO₂ fixation and O₂ release are estimated based on the photosynthetic reaction [28,29]: 6CO₂ + 6H₂O (light, enzymes, chloroplasts) → (CH₂O)ₙ + 6O₂

From this reaction, it can be derived that the production of 1 g of dry matter fixes 1.63 g of CO₂ and releases 1.2 g of O₂. That is, each gram of NPP corresponds to 1.63 g of CO₂ sequestration and 1.2 g of O₂ release. The value of carbon sequestration and oxygen release is calculated as follows:


$$\begin{array}{c} V_{B3}=V_{B3a}+V_{B3b} \end{array}$$ (8)

$$\begin{array}{c} V_{B3a}=1.63\;\times \;NPP\;\times \;Ctr\;\times \;S \end{array}$$ (9)

$$\begin{array}{c} V_{B3b}=\;1.2\;\times \;NPP\;\times \;Cop\;\times \;S \end{array}$$ (10)

In the equation, V_B_₃ denotes the total value of carbon sequestration and oxygen release; V_B_₃_a_ represents the value of carbon sequestration; V_B3b_ represents the value of oxygen release; Ctr denotes the carbon tax rate, taken as the average value since the initial implementation of carbon taxation in China (approximately 30 CNY/t), calculated based on data from the China Investment Association, http://www.iac.org.cn/default.asp?channel_id=81&info_id=82163&pg=23; The Ministry of Ecology and Environment of China, https://www.caep.org.cn/yclm/zghjghyzjzs/zghjghyzjzs_21956/202106/t20210605_836403.shtml; and the Shandong Provincial Development and Reform Commission， http://fgw.shandong.gov.cn/art/2023/1/4/art_208019_10379108.html. Cop represents the industrial oxygen production cost (400 CNY/t) [26]; and S denotes the grassland area.

(5) Air Purification

Grassland ecosystems purify the atmosphere by absorbing and degrading pollutants such as sulfur dioxide and fluorides, as well as by intercepting and adsorbing particulate matter. This study employs the market value method to estimate the economic value of atmospheric purification by grassland ecosystems [26]. The formula is as follows:


$$\begin{array}{c} V_{B4}=S\times (K_{SO2}\times P_{SO2})+S\times (K_{d}\times P_{d}) \end{array}$$ (11)

In the equation, V_B_₄ represents the value of air purification; S denotes the grassland area; K_SO₂_ is the purification efficiency for SO₂; P_SO₂_ is the market price for SO₂ purification; K_d_ represents the purification efficiency for particulate matter; and P_d_ is the market price for dust treatment. According to existing studies, grassland absorbs 21.7 kg·hm ⁻ ² of SO₂ [30] and intercepts 0.12 t·hm ⁻ ² of particulate matter [31].

### 2.2 Inter-industry ecological compensation allocation model

(1) Livestock Ecological Compensation Allocation Model

The ecological compensation for direct products of grasslands mainly targets the compensation for the consumption of grassland ecosystem products by the local livestock industry. Specifically, the consumption of grassland direct products by the livestock industry is represented by the actual consumption of beef and mutton, while the consumption of grassland indirect products mainly involves carbon sequestration and oxygen release. The formula is as follows:


$$\begin{array}{c} {Con}_{A1}=G\times U_{A} \end{array}$$ (12)

$$\begin{array}{c} {Con}_{A2}=Ctr\times U_{{ACO}_{2}}+Cop\times U_{{AO}_{2}} \end{array}$$ (13)

$$\begin{array}{c} {Com}_{A1}=V_{A}-{Con}_{A1} \end{array}$$ (14)

$$\begin{array}{c} {Com}_{A2}=V_{B3}\times \theta_{1}-{Con}_{A2} \end{array}$$ (15)

In the equation: Con_A1_ represents the value of actual consumption of grassland direct products by the livestock industry. G denotes the unit price of sheep. U_A_ represents the actual output of sheep. Con_A2_ represents the value of carbon sequestration and oxygen release consumed by the livestock industry. U_Aco2_ represents the carbon dioxide emissions from the livestock industry. U_Ao2_ represents the oxygen consumption by the livestock industry. Com_A1_ represents the ecological compensation amount for the direct products of grassland by the livestock industry. Com_A2_ represents the ecological compensation amount for the indirect products of grassland by the livestock industry. If Com > 0, it indicates that the industry does not need to bear ecological compensation, and this value reflects the compensation surplus. If Com < 0, it indicates that the industry needs to bear the corresponding ecological compensation. θ₁ represents the proportion of the livestock industry in the total output value of the livestock, industrial, and tourism sectors.

(2) Industrial Ecological Compensation Allocation Model

The ecological compensation for indirect products of grasslands primarily comes from the compensation for the consumption of grassland indirect products during the development of industry and tourism. The consumption of grassland indirect products by industry mainly includes water conservation, carbon sequestration and oxygen release, and air purification. The formula is as follows:


$$\begin{array}{c} {Con}_{B1}=Src\times U_{BSe} \end{array}$$ (16)

$$\begin{array}{c} {Con}_{B3}=Ctr\times U_{B{CO}_{2}}+Cop\times U_{{BO}_{2}} \end{array}$$ (17)

$$\begin{array}{c} {Con}_{B4}=K_{{SO}_{2}}\times U_{{BSO}_{2}}+K_{d}\times U_{Bd} \end{array}$$ (18)

$$\begin{array}{c} {Com}_{B1}=V_{B1}\times \theta_{2}-{Con}_{B1} \end{array}$$ (19)

$$\begin{array}{c} {Com}_{B3}=V_{B3}\times \theta_{3}-{Con}_{B3} \end{array}$$ (20)

$$\begin{array}{c} {Com}_{B4}=V_{B4}-{Con}_{B4} \end{array}$$ (21)

In the equation: Con_B1_ represents the value of water conservation consumed by industry. U_BSe_ represents the industrial wastewater discharge. Con_B3_ represents the value of carbon sequestration and oxygen release consumed by industry. U_Bco2_ represents the carbon dioxide emissions from the industrial sector. U_Bo2_ represents the oxygen consumption in the industrial sector. Con_B4_ represents the value of air purification consumed by industry. U_Bso2_ represents the sulfur dioxide emissions from the industrial sector. U_Bd_ represents the particulate matter emissions from the industrial sector. Com_B1_ represents the ecological compensation for water conservation provided by industry. Com_B3_ represents the ecological compensation for carbon sequestration and oxygen release provided by industry. Com_B4_ represents the ecological compensation for air purification provided by industry. θ₂ represents the proportion of industry in the total output value of industry and tourism. θ₃ represents the proportion of industry in the total output value of livestock, industry, and tourism.

(3) Tourism Ecological Compensation Allocation Model

The consumption of grassland indirect products by the tourism industry mainly involves water conservation, soil retention, and carbon sequestration and oxygen release. The formula is as follows:


$$\begin{array}{c} {Con}_{C1}=Src\times \text{Tr }\times \text{L} \end{array}$$ (22)

$$\begin{array}{c} {Con}_{C2}=WL\times ASE\times \alpha \end{array}$$ (23)

$$\begin{array}{c} {Con}_{C3}=Ctr\times U_{D{CO}_{2}}+Cop\times U_{{DO}_{2}} \end{array}$$ (24)

$$\begin{array}{c} {Com}_{C1}=V_{B1}\times \theta_{4}-{Con}_{C1} \end{array}$$ (25)

$$\begin{array}{c} {Com}_{C2}=V_{B2}-{Con}_{C2} \end{array}$$ (26)

$$\begin{array}{c} {Com}_{C3}=V_{B3}\times \theta_{5}-{Con}_{C3} \end{array}$$ (27)

In the equation: Con_C1_ represents the value of water conservation consumed by the tourism industry. Tr represents tourism intensity. L represents the per capita domestic wastewater discharge, with a value of 0.17 t per person [32]. Con_C2_ represents the value of soil retention consumed by the tourism industry. ASE represents the actual soil erosion. α represents the weight of tourism activities on actual soil erosion, calculated by the entropy weight method with a value of 0.44 (the calculation process is detailed below). Con_C3_ represents the value of carbon sequestration and oxygen release consumed by the tourism industry. U_Dco2_ represents the carbon dioxide emissions from tourism. U_Do2_ represents the oxygen consumption by tourism. Com_C1_ represents the ecological compensation for water conservation provided by tourism. Com_C2_ represents the ecological compensation for soil retention provided by tourism. Com_C3_ represents the ecological compensation for carbon sequestration and oxygen release provided by tourism. θ₄ represents the proportion of tourism in the total output value of industry and tourism. θ₅ represents the proportion of tourism in the total output value of livestock, industry, and tourism.

The calculation process of α is as follows. Based on existing studies [33,34], the weights of tourism intensity, precipitation, temperature, and grassland area on soil erosion are calculated using the entropy weight method. The formula is as follows:


$$\begin{array}{c} P_{ij}=\frac{y_{ij}}{\sum_{i=1}^{m}y_{ij}} \end{array}$$ (28)

$$\begin{array}{c} e_{j}=-k\sum_{i=1}^{m}P_{ij}{lnP}_{ij},k>0,0\leq e_{ij}\leq 1 \end{array}$$ (29)

$$\begin{array}{c} g_{i}=1-e_{ij} \end{array}$$ (30)

$$\begin{array}{c} \alpha_{j}=\frac{g_{i}}{\sum_{j=1}^{n}g_{j}} \end{array}$$ (31)

In the equation: αⱼ represents the weight of each influencing factor on soil erosion in grasslands. Based on the calculation, the weight for tourism intensity is 0.44, precipitation is 0.35, temperature is 0.16, and grassland area is 0.04.

### 2.3 Data sources

The data for livestock production, livestock output value, industrial output value, tourism numbers, and tourism output value in this study are sourced from the China Statistical Yearbook (2013–2023) [https://tj.nmg.gov.cn/datashow/pubmgr/publishmanage.htm?m=queryPubData&procode=0003, https://tjj.xinjiang.gov.cn/tjj/zhhvgh/list_nj1.shtml, http://tjj.qinghai.gov.cn/nj/2022/lefte.htm]. The data on livestock product prices are obtained from the China Animal Husbandry and Veterinary Yearbook (2013–2023) [https://www.nahs.org.cn/jchsjcm/]. The cost of reservoir construction is taken from the China Water Resources Yearbook (2013–2023) [http://www.mwr.gov.cn/]. The grassland area and annual average grassland yield data are sourced from the China Forestry and Grassland Statistical Yearbook (2013–2023) [http://202.99.63.178/c/www/tjnj.jhtml]. Precipitation data are obtained from the ERA5-Land dataset published by organizations such as the European Union and the European Centre for Medium-Range Weather Forecasts (https://cds.climate.copernicus.eu/cdsapp#!/dataset/reanalysis-era5-land-monthly-means?tab=overview‌‌). Evapotranspiration data come from the National Qinghai-Tibet Plateau Data Center (https://data.tpdc.ac.cn/home). The NPP data are sourced from the NASA Earth Science Data website (https://search.earthdata.nasa.gov/). SO_2_ and dust treatment cost data are obtained from the China Ecological Environment Statistical Yearbook (2013–2023) [https://www.mee.gov.cn/hjzl/sthjzk/sthjtjnb/].

## 3. Research results

### 3.1 Grassland ecosystem product value supply

(1) Direct Products

**Fig 2:**
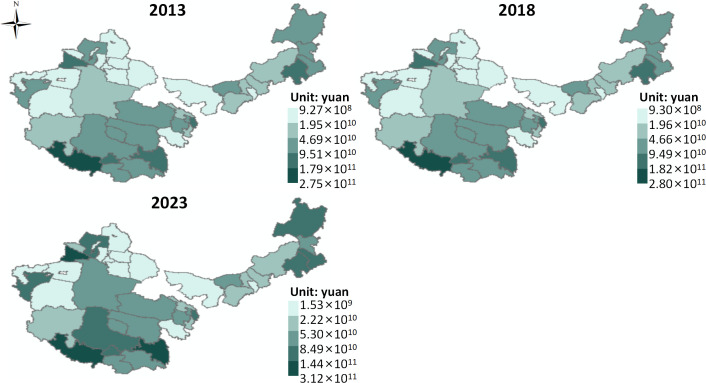
Value supply of direct ecological products. Map quoted from the National Geospatial Information Research Directory Service System - www.webmap.cn. Map Approval Number GS(2024)0650.

The supply of direct grassland ecosystem product values (Fig 2). On the whole, from 2013 to 2023, the average supply of direct ecosystem product value in the four major pastoral regions increased from 5.79 × 10¹⁰ CNY to 6.84 × 10¹⁰ CNY. This indicates an upward trend in the supply of direct ecosystem product value in the four pastoral regions, which could be attributed to the significant improvement in total factor productivity within the livestock production function, thus increasing resource output efficiency per unit of input [35]. Meanwhile, institutional arrangements such as ecological compensation, grazing bans, and grass-livestock balance have optimized herder behavior through a “constraint-incentive” mechanism, achieving a dynamic balance between ecological protection and production efficiency [36]. In addition, the rise in livestock product prices and quality premiums, under the backdrop of consumption upgrades, further amplified the market effect of value growth. Furthermore, grassland ecological restoration improved vegetation coverage and ecological carrying capacity, providing a more stable production foundation for livestock farming.

Using the natural break classification method, the spatial distribution of direct ecosystem product value supply is divided into five levels, from high to low, including higher, high, medium, low, and lower levels. From 2013 to 2023, the spatial pattern in the four pastoral regions showed an evolution of “overall stability with partial changes.” Taking the spatial distribution in 2023 as an example, the overall pattern is “high in the southeast, low in the northwest.” Higher and high-level areas are mainly distributed in multiple block forms in the eastern part of Inner Mongolia, the northwestern part of Xinjiang, and the southwestern part of Tibet. Medium-level areas are mainly found in the southern part of Xinjiang, western Qinghai, and parts of eastern Tibet. Low and lower-level areas are mainly concentrated in the northwestern part of the study area.

It can be observed that the spatial pattern of direct ecosystem product value supply in the four major pastoral regions indicates a strong path dependency and resource-environmental constraint, which is also dynamically influenced by local production condition improvements and policy interventions. The “high in the southeast, low in the northwest” distribution characteristic observed in 2023 first reflects the spatial differences in the productivity of grassland ecosystems. Specifically, the regions with higher and high-level concentrations in eastern Inner Mongolia, northwestern Xinjiang, and southwestern Tibet have relatively concentrated grassland resources, favorable water-heat conditions, better vegetation productivity, or seasonal forage supply capacity, which provide a stronger material basis for livestock product production. Additionally, these regions tend to have well-established traditional pastoral development, mature livestock carrying and output systems, making it easier to realize the effective conversion of grassland ecological resources into economic value. In contrast, areas in southern Xinjiang, western Qinghai, and eastern Tibet, with medium-level concentrations, have some grassland supply capacity but are constrained by ecological vulnerability, fragmented resource space, or infrastructure limitations [37], and their value realization ability has not reached a high concentration level. The concentration of low and lower-level areas in the northwestern part of the study area indicates that factors such as deepening arid conditions, harsh ecological environments, low grassland productivity, and unfavorable transportation and location have significantly suppressed the direct ecosystem product value. It is worth noting that although the spatial pattern has not undergone a fundamental reconstruction over the ten years, local changes indicate that the supply of direct ecosystem product values is not entirely determined by static natural conditions but is also influenced by a certain degree of plasticity.

(2) Indirect Products

**Fig 3:**
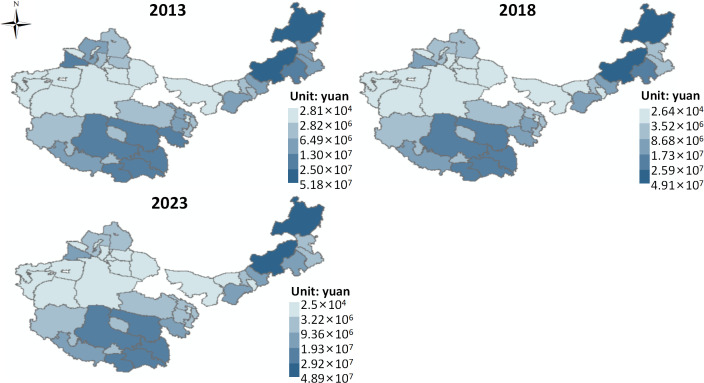
Value supply of water conservation. Map quoted from the National Geospatial Information Research Directory Service System - www.webmap.cn. Map Approval Number GS(2024)0650.

The supply of grassland water conservation value (Fig 3). On the whole, from 2013 to 2023, the average supply of water conservation value in the four major pastoral regions remained stable at 9.68 × 10⁶ CNY. Using the natural break classification method, the spatial distribution of water conservation value supply is divided into five levels, from high to low, including higher, high, medium, low, and lower levels. Taking the spatial distribution in 2023 as an example, the overall pattern is “high in the east, low in the west.” Higher and high-level areas are mainly distributed in multiple block forms in eastern Inner Mongolia, southern Qinghai, and eastern Tibet. Medium-level areas are mainly distributed in central Inner Mongolia, northern Xinjiang, northern Qinghai, and parts of western Tibet. Low and lower-level areas are mainly distributed in the northwestern part of the study area.

It can be observed that the water conservation value supply in the four major pastoral regions from 2013 to 2023 remained generally stable, and the spatial pattern exhibited a characteristic of “overall stability, high in the east, low in the west,” reflecting the high dependence of water conservation functions on natural geographic conditions and their relative insensitivity to human disturbance. From a formation mechanism perspective, water conservation, as a typical regulating ecosystem service, mainly depends on natural factors such as precipitation input, vegetation coverage, soil structure, and topography [38], rather than being directly driven by market and production behaviors. As a result, fluctuations in its temporal dimension are relatively small, exhibiting significant rigid supply characteristics. Spatially, the “high in the east, low in the west” pattern is primarily driven by the precipitation gradient, with eastern Inner Mongolia, southern Qinghai, and eastern Tibet significantly influenced by monsoon precipitation, resulting in relatively abundant rainfall, high vegetation coverage, and better soil organic matter content, thus significantly enhancing water conservation capacity through interception, infiltration, and retention processes. In contrast, regions like northern Xinjiang, northern Qinghai, and western Tibet, with medium-level concentrations, possess certain precipitation conditions or support from cold grassland ecosystems but are limited by climate fluctuations, topographic undulations, and vegetation structure, placing their water conservation capacity at a medium level. The concentration of low and lower-level areas in the northwestern part of the study area is primarily due to factors such as arid climate, scarce precipitation, intense evaporation, and sparse vegetation, resulting in significantly insufficient water retention and soil water-holding capacity, thus restricting the water conservation function [39]. This spatial pattern has remained stable during the study period, indicating a clear natural baseline effect of water conservation, but local differences may also be influenced by ecological restoration projects and land use adjustments at the margin.

**Fig 4:**
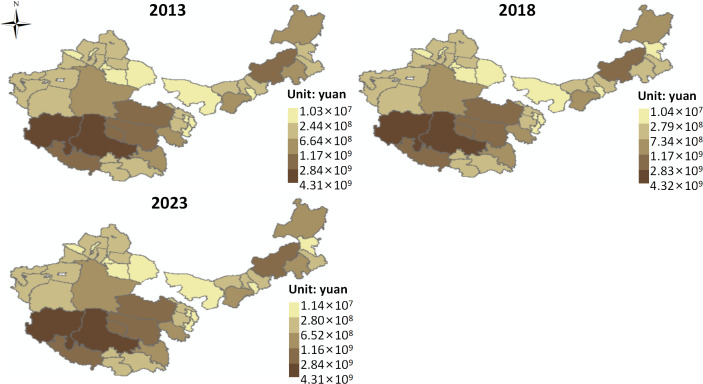
Value supply of soil conservation in grassland. Map quoted from the National Geospatial Information Research Directory Service System - www.webmap.cn. Map Approval Number GS(2024)0650.

The supply of grassland soil conservation value (Fig 4). On the whole, from 2013 to 2023, the average supply of soil conservation value in the four major pastoral regions decreased slightly from 8.14 × 10⁸ CNY to 8.12 × 10⁸ CNY. Using the natural break classification method, the spatial distribution of soil conservation value supply is divided into five levels, from high to low, including higher, high, medium, low, and lower levels. Taking the spatial distribution in 2023 as an example, the overall pattern is “high in the south, low in the north.” Higher and high-level areas are mainly distributed in a single block in northwestern Tibet and northwestern Qinghai. Medium-level areas are mainly distributed in central and eastern Inner Mongolia, southern Xinjiang, southeastern Qinghai, and southeastern Tibet. Low and lower-level areas are mainly distributed in the central and northwestern parts of the study area.

From 2013 to 2023, the supply of grassland soil conservation value in the four major pastoral regions showed a slight downward trend, which could be due to the fact that soil conservation, as a typical regulating ecosystem service, depends heavily on natural control factors such as precipitation erosivity, topography, vegetation coverage, and soil erosion resistance. Consequently, the overall fluctuation is relatively small, but the slight decrease may reflect the combined effects of grassland degradation, overgrazing, or extreme climate events in certain areas, which have led to an increased risk of soil erosion and thus weakened the overall soil conservation function. Spatially, the higher and high-level areas in northwestern Tibet and northwestern Qinghai mainly arise due to the large topographic undulations and steep slopes in the high-altitude cold regions, where the relatively good vegetation cover enhances the soil erosion interception and fixation effect, leading to higher soil conservation value per unit area. Additionally, these areas experience relatively less human disturbance, allowing the ecosystems to maintain strong natural regulatory functions. Medium-level areas in central and eastern Inner Mongolia, southern Xinjiang, and the transitional zone of the Qinghai-Tibet Plateau indicate that while these areas possess certain vegetation cover and topographic conditions, they are also influenced by human factors such as grazing intensity and land use changes, causing the soil conservation function to remain relatively balanced but unstable. In contrast, the concentration of low and lower-level areas in the central and northwestern parts of the study area is primarily due to unfavorable natural conditions such as arid climate, wind and water erosion, sparse vegetation, and fragile soil structure, coupled with human pressures such as overgrazing, leading to weaker soil conservation capacity. The spatial pattern has remained generally stable during the study period, but the slight downward trend suggests that this function is sensitive to ecological degradation, and marginal changes in human utilization patterns may trigger cumulative losses in ecosystem services.

**Fig 5:**
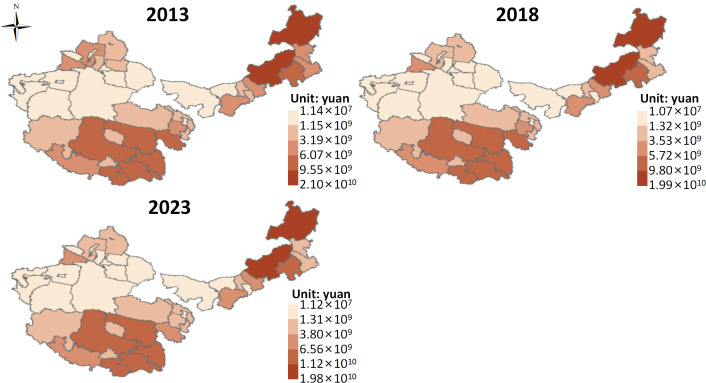
Value supply of carbon sequestration and oxygen release in grassland. Map quoted from the National Geospatial Information Research Directory Service System - www.webmap.cn. Map Approval Number GS(2024)0650.

Grassland Carbon Sequestration and Oxygen Release Value Supply (Fig 5). On the whole, from 2013 to 2023, the average carbon sequestration and oxygen release value supply in the four major pastoral regions slightly decreased from 3.84 × 10⁹ CNY to 3.83 × 10⁹ CNY. Using the natural break classification method, the spatial distribution of carbon sequestration and oxygen release value supply is divided into five levels, from high to low, including higher, high, medium, low, and lower levels. Taking the spatial distribution in 2023 as an example, the overall pattern is “high in the southeast and south, low in the northwest and north.” Higher and high-level areas are mainly distributed in multiple block forms in eastern Inner Mongolia, southern Qinghai, and southeastern Tibet. Medium-level areas are mainly found in central Inner Mongolia, northern Xinjiang, northern Qinghai, and parts of western Tibet. Low and lower-level areas are mainly concentrated in the central and northwestern regions of the study area.

It can be observed that the carbon sequestration and oxygen release value supply in the four major pastoral regions has shown a slight decline from 2013 to 2023, reflecting the dynamic balance of the carbon sink function of the grassland ecosystem under the joint action of natural constraints and human disturbances. As a regulatory ecosystem service dependent on vegetation photosynthesis, the supply level of carbon sequestration and oxygen release mainly depends on net primary productivity (NPP), community structure, and biomass accumulation, which have a strong natural control attribute. Thus, the overall fluctuation is limited [40]. However, the observed declining trend suggests that some areas may have experienced grassland degradation, overgrazing, or climate change, which have suppressed vegetation productivity and carbon fixation ability.

From a spatial pattern perspective, the distribution characteristics of carbon sequestration and oxygen release value supply reflect the combined effects of water-heat conditions, vegetation types, and gradients of ecosystem productivity. The higher and high-level concentrated areas in eastern Inner Mongolia, southern Qinghai, and southeastern Tibet are primarily due to strong monsoon influences in these regions, resulting in relatively abundant rainfall, favorable temperature conditions, high vegetation or alpine meadow coverage, and high biomass, leading to a high capacity for carbon sequestration and oxygen release. Additionally, these areas have strong ecological protection, which helps maintain higher levels of ecosystem functions. The medium-level areas, located in central Inner Mongolia, northern Xinjiang, and the transitional zone of the Qinghai-Tibet Plateau, indicate that these regions are in a transitional state in terms of water-heat conditions and vegetation productivity, with relatively stable but not exceptional carbon fixation capacity. The low and lower-level areas in the central and northwestern parts of the study area are mainly restricted by factors such as arid climate, insufficient precipitation, intense evaporation, and sparse vegetation, resulting in low NPP and a significantly weak carbon sink function.

**Fig 6:**
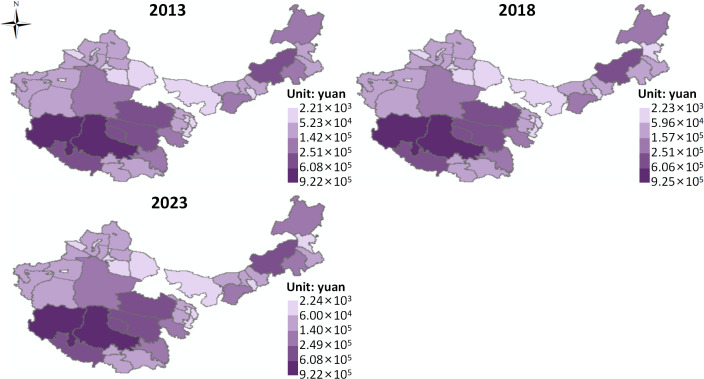
Value supply of air purification in grassland. Map quoted from the National Geospatial Information Research Directory Service System - www.webmap.cn. Map Approval Number GS(2024)0650.

Grassland Air Purification Value Supply (Fig 6). On the whole, from 2013 to 2023, the average air purification value supply in the four major pastoral regions slightly decreased from 1.75 × 10⁵ CNY to 1.72 × 10⁵ CNY. Using the natural break classification method, the spatial distribution of air purification value supply is divided into five levels, from high to low, including higher, high, medium, low, and lower levels. Taking the spatial distribution in 2023 as an example, the overall pattern is “high in the south, low in the north.” Higher and high-level areas are mainly distributed in a single block form in northwestern Tibet and western Qinghai. Medium-level areas are mainly found in eastern Inner Mongolia, southern Xinjiang, southeastern Qinghai, and parts of eastern Tibet. Low and lower-level areas are primarily concentrated in the northwestern part of the study area.

From 2013 to 2023, the air purification value supply in the four major pastoral regions showed a slight decline but remained relatively stable, indicating that the air purification function has a certain buffering capacity against external disturbances under the dominance of natural constraints, though there is still a marginal weakening trend. The air purification function mainly relies on vegetation’s ability to adsorb, absorb, and settle atmospheric pollutants through leaf area index (LAI), stomatal conductance, and surface roughness. Its supply capacity is directly controlled by vegetation coverage, biomass, and community structure. Therefore, the slight overall decrease reflects the negative impacts of grassland degradation, vegetation sparsity, or climate change on vegetation growth, thereby weakening the interception and purification capacity for pollutants.

From a spatial pattern perspective, higher and high-level areas are mainly concentrated in northwestern Tibet and western Qinghai. Despite the harsh climate in some areas, the alpine grassland vegetation in specific topographies and local water-heat conditions has strong coverage and ecological stability, with relatively weak human disturbance and a better atmospheric environment, resulting in more prominent air purification services per unit area. Medium-level areas are distributed in eastern Inner Mongolia, southern Xinjiang, and the transitional zone of the Qinghai-Tibet Plateau, indicating that these areas have medium-level vegetation conditions and ecological functions. While they have some purification capacity, they are influenced by climate fluctuations and grazing activities. The low and lower-level areas concentrated in the northwestern part of the study area are primarily restricted by factors such as arid climate, sparse vegetation, strong winds, and frequent dust storms, which not only reduce the vegetation’s ability to intercept pollutants but also exacerbate the concentration of particulate matter in the air, thus weakening the air purification function.

### 3.2 Grassland ecological compensation

(1) Ecological Compensation for Livestock

**Fig 7:**
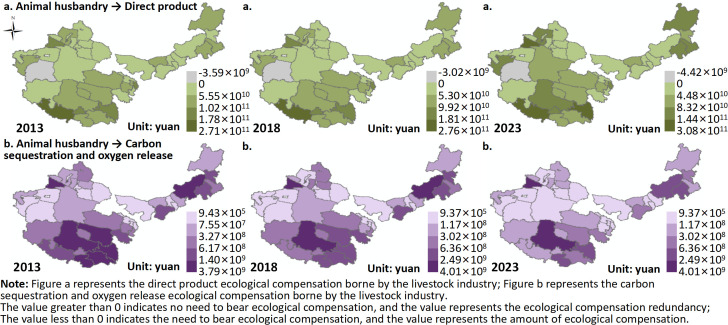
Ecological compensation in animal husbandry. Map quoted from the National Geospatial Information Research Directory Service System - www.webmap.cn. Map Approval Number GS(2024)0650.

This study primarily calculates the ecological compensation for livestock concerning direct grassland products and carbon sequestration and oxygen release. The ecological compensation for direct grassland products by livestock (Fig 7). From 2013 to 2023, in most areas, the ecological compensation for direct grassland products by livestock was greater than 0, indicating that most regions do not need to bear this part of the ecological compensation. The average compensation surplus increased from 5.50 × 10¹⁰ CNY to 6.55 × 10¹⁰ CNY. In contrast, from 2013 to 2023, the ecological compensation for direct grassland products in the Hetian region of Xinjiang was less than 0, indicating that this area is required to bear ecological compensation, with compensation increasing from 3.59 × 10⁹ CNY to 4.42 × 10⁹ CNY.

In terms of spatial distribution, the ecological compensation surplus for direct grassland products from 2013 to 2023 shows an “overall low at the center, high at the edges” spatial pattern. For instance, in 2023, areas with high ecological compensation surplus are mainly concentrated in the Ili region, Tacheng, Kashgar, Shigatse, Chamdo, Naqu in Tibet, Hohhot, Tongliao, and Chifeng in Inner Mongolia. Low surplus areas are mainly found in central Inner Mongolia, eastern Xinjiang, and eastern Qinghai.

From the above, it can be concluded that from 2013 to 2023, in most regions of the four major pastoral areas, the ecological compensation for direct grassland products by livestock is greater than 0, and the average compensation surplus shows an upward trend. This indicates that, on the whole, livestock production has a strong supporting effect on the realization of the value of direct grassland products, and most regions have not exceeded the supply boundary of the grassland ecosystem, thus not requiring additional compensation for this ecological loss. This reflects a generally coordinated state between the supply of direct grassland products and livestock pressure. On one hand, areas such as eastern Inner Mongolia, western Xinjiang, and parts of Tibet, with better grassland resources, a stronger livestock foundation, and higher grass-livestock conversion efficiency, can achieve high livestock product values with relatively low ecological pressure, leading to large ecological compensation surpluses. On the other hand, advancements in breeding, forage supply, semi-pastoral systems, and disease control have improved the output efficiency per unit of grassland resources, making livestock growth more reliant on productivity improvements rather than simply expanding grassland consumption, thus increasing the surplus of ecological compensation. Meanwhile, policies such as grassland ecological protection subsidies, grazing bans, and grass-livestock balance have controlled extensive utilization patterns, further solidifying the ecological compensation surplus in most regions.

In contrast, the ecological compensation for direct grassland products in the Hetian region of Xinjiang remained less than 0 during 2013–2023, with the compensation amount increasing, indicating that livestock development in this region may be causing a net consumption of direct grassland products. This suggests some ecological overdraft, reflecting structural contradictions between fragile ecological foundations, low grassland productivity, strong resource-environmental constraints, and mismatched livestock utilization intensity [41].

From a spatial distribution perspective, the overall pattern of ecological compensation surplus for direct grassland products shows “low in the center—high at the edges,” indicating that the core transitional zones often face higher population, production, and resource utilization pressures, making it more likely for the supply of ecological products and livestock demand to reach a balanced state. In contrast, the peripheral areas, especially those such as the Ili region in Xinjiang, Tacheng, Kashgar, Shigatse, Chamdo, Naqu in Tibet, and Hohhot, Tongliao, Chifeng in Inner Mongolia, where high compensation surplus areas form, reflect the relatively better grassland resource base and higher coupling between livestock farming and the ecosystem, allowing the ecological product supply to effectively meet the economic demand.

The ecological compensation for livestock in terms of carbon sequestration and oxygen release in grasslands (Fig 7). From 2013 to 2023, the ecological compensation for livestock’s carbon sequestration and oxygen release in the four major pastoral regions was greater than 0, indicating that no additional compensation is required for this aspect. However, the average ecological compensation surplus decreased from 7.38 × 10⁸ CNY to 7.09 × 10⁸ CNY.

In terms of spatial distribution, the ecological compensation surplus for carbon sequestration and oxygen release shows an “overall high in the southeast and east, low in the northwest” pattern from 2013 to 2023. In 2023, high compensation surplus areas are mainly distributed in the Ili region of Xinjiang, Naqu, Chamdo, Shannan in Tibet, Xilin Gol League in Inner Mongolia, and Chifeng. Low surplus areas are mainly found in western Inner Mongolia, central Xinjiang, and eastern Qinghai.

From 2013 to 2023, the ecological compensation for carbon sequestration and oxygen release by livestock in the four major pastoral regions showed a decreasing trend in compensation surplus, indicating that livestock activities have not yet exceeded the carbon sink function’s capacity of grassland ecosystems, and the carbon sequestration and oxygen release function is still effectively absorbing the carbon emission pressure generated by livestock production. However, the surplus space for ecological compensation is shrinking. This decline reflects the fact that under the continuous development of livestock farming, the rate of growth of carbon emissions is approaching or even surpassing the increase in the carbon sequestration capacity of grassland ecosystems, indicating that the balance between carbon sources and sinks is tightening. This suggests a cumulative response to human activities and climate change, where, under relatively stable natural conditions, the marginal increase in livestock development may gradually erode the ecological system’s carbon sink safety margin [42].

This change is likely due to grassland degradation, decreased vegetation cover, and the suppression of carbon fixation ability by climate change in some areas, as well as the increased carbon emissions from the expansion of livestock farming and intensified production activities. Spatially, the carbon sequestration and oxygen release ecological compensation surplus shows a “high in the southeast and east, low in the northwest” distribution pattern, reflecting the spatial differentiation of climatic conditions and vegetation productivity. High surplus areas such as the Ili region in Xinjiang, Naqu, Chamdo, Shannan in Tibet, and Xilin Gol League and Chifeng in Inner Mongolia have relatively favorable water-heat conditions or support from alpine meadow ecosystems, high vegetation cover, and strong biomass, enabling them to absorb carbon emissions from livestock farming, thus forming high ecological compensation surpluses. On the other hand, low surplus areas in western Inner Mongolia, central Xinjiang, and eastern Qinghai are limited by factors such as arid climate, insufficient precipitation, and sparse vegetation, which lead to low NPP and limited carbon sequestration capacity. Additionally, some grazing pressure further exacerbates the carbon source-sink relationship, leading to smaller ecological compensation surplus.

(2) Industrial Ecological Compensation

**Fig 8:**
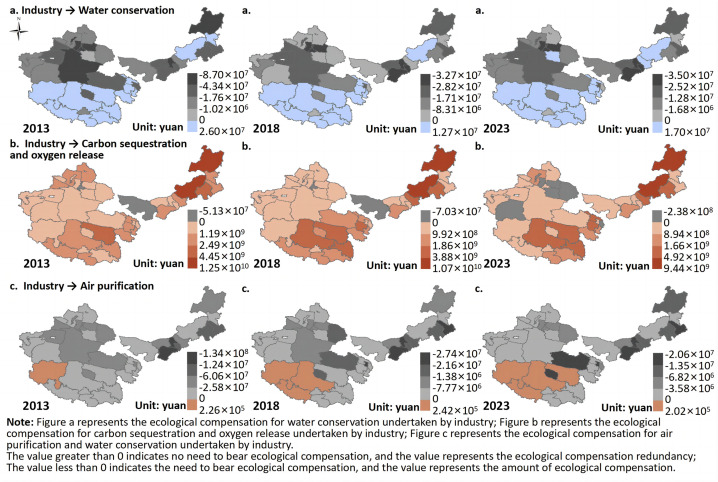
Ecological compensation in industry. Map quoted from the National Geospatial Information Research Directory Service System - www.webmap.cn. Map Approval Number GS(2024)0650.

This study primarily calculates the ecological compensation for the industrial sector concerning grassland water conservation, carbon sequestration and oxygen release, and air purification. The ecological compensation for grassland water conservation by industry (Fig 8). From 2013 to 2023, areas where the ecological compensation for grassland water conservation was greater than 0 were mainly concentrated in the southern regions of Qinghai and Tibet, with the average ecological compensation surplus decreasing from 7.36 × 10⁶ CNY to 7.15 × 10⁶ CNY. In contrast, areas where the ecological compensation was less than 0 were mainly concentrated in the northern regions of Inner Mongolia and Xinjiang, with the required average compensation decreasing from 2.39 × 10⁷ CNY to 1.23 × 10⁷ CNY.

In terms of spatial distribution, the ecological compensation for grassland water conservation from 2013 to 2023 shows a “west to east shift” spatial pattern. In 2023, high ecological compensation areas were primarily found in Hohhot, Ordos, Urumqi, and Changji in Xinjiang, while low compensation areas were mainly distributed in eastern Inner Mongolia and parts of northern Xinjiang.

From 2013 to 2023, the ecological compensation for grassland water conservation exhibited significant regional differentiation and dynamic adjustment characteristics, reflecting a spatial mismatch between industrial water demand and the grassland water regulation function, and its evolution over time. On the whole, the southern regions like Qinghai and Tibet had positive compensation amounts, but with declining surpluses, indicating that these areas have strong water conservation functions and relatively low industrial development intensity. The grassland ecosystem’s supply capacity can cover industrial water pressure, but the surplus space has slightly contracted, reflecting a weakened safety margin for water regulation due to increasing industrial activity or climate fluctuations. In contrast, northern regions like Inner Mongolia and Xinjiang had negative ecological compensation amounts for water conservation, but the pressure has eased over time, possibly due to advancements in water-saving technologies, industrial restructuring, and improved water efficiency. Spatially, the “west to east shift” pattern reflects the migration of industrial water pressure and its ecological impact, influenced by changes in regional economic focus and resource-environment constraints [43]. The high compensation areas in Hohhot, Ordos, Urumqi, and Changji, which are industrial hubs with a large proportion of energy and chemical industries, have high water demand and limited water conservation capacity, thus exerting significant pressure on the grassland ecosystem. On the other hand, low-compensation areas in eastern Inner Mongolia and northern Xinjiang, which have relatively better precipitation or lower industrial intensity, maintain a relatively balanced relationship between water conservation function and water demand.

The ecological compensation for carbon sequestration and oxygen release by industry (Fig 8). From 2013 to 2023, in most areas, the ecological compensation for carbon sequestration and oxygen release by industry was greater than 0, with the average ecological compensation surplus decreasing from 1.64 × 10⁹ CNY to 1.29 × 10⁹ CNY. Areas where compensation was negative were mainly located in some regions of Inner Mongolia and Xinjiang, with the required average compensation increasing from 2.50 × 10⁷ CNY to 9.38 × 10⁷ CNY.

In terms of spatial distribution, the ecological compensation surplus for carbon sequestration and oxygen release shows an “east high, west low” spatial pattern from 2013 to 2023. In 2023, high ecological compensation surplus areas were primarily found in Hohhot, Xilin Gol League, Haibei and Hainan in Qinghai, Naqu and Chamdo in Tibet, while low surplus areas were mainly located in western Inner Mongolia and most parts of Xinjiang. Meanwhile, from 2013 to 2023, the spatial pattern of ecological compensation for carbon sequestration and oxygen release shifted from “east to west,” with the number of regions requiring compensation increasing from 3 to 7.

It can be observed that from 2013 to 2023, the ecological compensation for carbon sequestration and oxygen release by industry was predominantly positive, but the average compensation surplus decreased, while the number of regions requiring compensation increased significantly. This suggests that the pressure on grassland carbon sink functions from industrial development is gradually increasing, reflecting a structural change where carbon source expansion outpaces the enhancement of carbon sinks. Areas in Inner Mongolia and Xinjiang that shifted from positive to negative compensation and showed an increase in compensation amounts indicate that industrial carbon emissions in these areas have exceeded the carbon sequestration capacity of the grassland ecosystem, causing the system to shift from a net carbon sink to a net deficit. This reflects the increasing contradiction between energy-intensive industrial clusters and fragile ecological foundations. From a spatial pattern perspective, the ecological compensation surplus for carbon sequestration and oxygen release exhibits an “east high, west low” distribution, while the ecological compensation pressure shows a “westward shift.” This reveals the asymmetry between carbon sink capacity and industrial emissions [44], with higher surplus areas in places like Hohhot, Xilin Gol League, and parts of Qinghai and Tibet benefiting from better vegetation productivity and lower industrial intensity, thus maintaining a stronger carbon sink function. In contrast, areas in western Inner Mongolia and most of Xinjiang, which are experiencing carbon deficits, face challenges due to sparse vegetation, limited carbon fixation capacity, and high carbon emissions from energy and heavy industry.

The ecological compensation for air purification by industry (Fig 8). From 2013 to 2023, in most areas, the ecological compensation for air purification by industry was negative, with the average ecological compensation amount decreasing from 2.76 × 10⁷ CNY to 4.44 × 10⁶ CNY. Positive compensation areas were mainly located in parts of Tibet and Qinghai, with the average ecological compensation surplus decreasing from 2.25 × 10⁵ CNY to 1.08 × 10⁵ CNY.

In terms of spatial distribution, the ecological compensation for air purification shows an “east high, west low” spatial pattern from 2013 to 2023. In 2023, high compensation areas were mainly found in Tongliao, Baotou, and Ordos in Inner Mongolia, as well as Xining and Haixi in Qinghai. Low compensation areas were mainly distributed in most of Xinjiang and Tibet. Additionally, from 2013 to 2023, the ecological compensation surplus for air purification exhibited a “southeast to surrounding areas diffusion” spatial pattern, with surplus areas in 2023 concentrated in regions such as Ali, Naqu, Shigatse, and Yushu in Tibet and Qinghai.

From 2013 to 2023, the ecological compensation for air purification by industry was primarily negative, with a significant decrease in the average compensation amount and a continuous reduction in the size of positive surplus areas. This suggests that industrial emissions have created long-term and widespread net pressure on grassland air purification, although this pressure has somewhat eased.

The majority of regions with negative compensation amounts indicate that industrial pollution emissions have generally exceeded the grassland ecosystem’s self-cleaning capacity, creating an imbalance where pollution intensity exceeds the ecosystem’s ability to absorb it. The decline in compensation amounts may be due to the improvement of pollution control technologies, stricter emission standards, and industrial restructuring, reflecting the phased success of human interventions in alleviating ecological pressure. From a spatial pattern perspective, high compensation areas in places like Tongliao, Baotou, and Ordos, as well as Xining and Haixi in Qinghai, are industrial hubs with high emission intensity. These areas, combined with arid climate conditions and limited dispersion, lead to a higher accumulation of air pollutants, significantly increasing dependence on grassland air purification. In contrast, low compensation areas in most of Xinjiang and Tibet, due to lower industrialization or larger atmospheric capacity, face relatively limited pollution pressure. At the same time, ecological compensation surpluses were mainly concentrated in Tibet’s Ali, Naqu, Shigatse, and Yushu in Qinghai, indicating that these areas, with low industrial intensity, relatively intact ecological foundations, and good vegetation systems, still maintain some air purification capacity, though their surplus is decreasing, reflecting a weakening of their ecological buffering capacity.

(3) Ecological Compensation for the Tourism Industry

**Fig 9:**
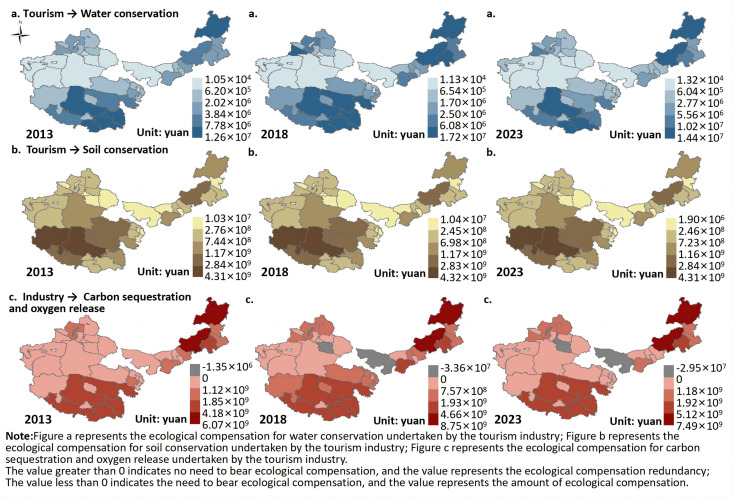
Ecological compensation in tourism industry. Map quoted from the National Geospatial Information Research Directory Service System - www.webmap.cn. Map Approval Number GS(2024)0650.

This study primarily calculates the ecological compensation borne by the tourism industry for grassland water conservation, soil retention, and carbon sequestration and oxygen release. The ecological compensation for grassland water conservation by tourism (Fig 9). From 2013 to 2023, the ecological compensation for grassland water conservation by tourism was greater than 0 across the four major pastoral regions, indicating that these regions did not need to bear this portion of the ecological compensation. The average ecological compensation surplus increased from 2.83 × 10⁶ CNY to 3.80 × 10⁶ CNY.

From a spatial distribution perspective, the ecological compensation surplus for water conservation exhibited an “overall high in the southeast and east, low in the northwest” pattern from 2013 to 2023. For example, in 2023, high surplus areas were primarily distributed in Hulunbuir and Xilin Gol League in Inner Mongolia, and in the cities of Shannan, Nyingchi, and Naqu in Tibet. Low surplus areas were mainly found in western Inner Mongolia and parts of central Xinjiang.

It can be observed that the ecological compensation for grassland water conservation by tourism from 2013 to 2023 was consistently positive, and the average compensation surplus increased, indicating that the tourism industry has not created a net consumption of grassland water conservation. On the contrary, it has shown a relatively coordinated or even positively reinforcing relationship with ecosystem service supply. In terms of mechanism, water conservation is primarily controlled by natural factors such as precipitation, vegetation coverage, and soil structure. As a relatively low-impact industry, tourism consumes resources at a lower intensity compared to industry and some livestock activities, resulting in limited direct disturbance to the water cycle. Furthermore, the development model of eco-tourism and nature conservation has, to some extent, enhanced vegetation quality and ecosystem stability through funding investment, ecological restoration, and environmental management, thereby indirectly increasing water conservation capacity. This is a key reason why the ecological compensation surplus has continued to grow.

The spatial distribution of the surplus (“high in the southeast and east, low in the northwest”) reflects the combined effect of natural water-heat conditions and the tourism development foundation. High-surplus areas in Hulunbuir, Xilin Gol League, and parts of Tibet, such as Shannan, Nyingchi, and Naqu, benefit from favorable precipitation conditions and high vegetation cover, providing strong water conservation capacity. Moreover, these regions have rich eco-tourism resources and a relatively standardized development model, which results in lower ecological pressure from tourism activities and even positive externalities. In contrast, low-surplus areas in western Inner Mongolia and central Xinjiang face constraints due to arid climates, water scarcity, and fragile ecological conditions, with limited water conservation capacity. Additionally, these areas have lower levels of tourism development or weak infrastructure, leading to smaller surplus space between ecological service supply and utilization.

The spatial pattern has remained generally stable, with surplus levels continuing to rise, suggesting that tourism has not surpassed the ecological carrying threshold for grassland water conservation. Instead, tourism has, to some extent, reinforced ecosystem service functions through “eco-dependent development.” This result indicates that compared to resource-consuming industries, tourism is more likely to achieve a positive coupling with grassland ecosystems, with its development path characterized by “low disturbance—high dependence—positive feedback.” However, it is important to note that as tourism scales up, its indirect demand for water resources may gradually increase, requiring future careful planning and capacity control to maintain the stability of ecological compensation surpluses and ensure the long-term coordination between tourism development and water conservation functions.

The ecological compensation for grassland soil retention by tourism (Fig 9). From 2013 to 2023, the ecological compensation for grassland soil retention by tourism remained positive across the four major pastoral regions, indicating that these regions did not need to bear this portion of the ecological compensation. However, the average compensation surplus decreased slightly from 7.98 × 10⁸ CNY to 7.87 × 10⁸ CNY.

From a spatial distribution perspective, the ecological compensation surplus for soil retention exhibited a “high in the south, low in the north” pattern from 2013 to 2023. For example, in 2023, high-surplus areas were mainly found in Ali and Naqu in Tibet, Xilin Gol League in Inner Mongolia, Yushu and Haixi in Qinghai, while low-surplus areas were mainly located in western Inner Mongolia and parts of eastern Xinjiang.

From 2013 to 2023, the ecological compensation for grassland soil retention by tourism remained positive, but the average surplus decreased slightly, indicating that tourism has not yet surpassed the ecological carrying threshold for grassland soil retention. However, the potential pressure on soil retention services is gradually becoming evident. Soil retention primarily depends on vegetation cover, root stabilization, and topographic conditions. Although tourism is a low-consumption industry overall, it can have marginal impacts on the surface structure and vegetation continuity through infrastructure construction, tourist trampling, and localized land disturbances, thus weakening the soil’s resistance to erosion. This impact tends to be “cumulative and localized,” so at the macro scale, it results in only a slight decrease in surplus. Nevertheless, it reflects a slow weakening trend in ecosystem regulatory functions.

The spatial pattern shows that high-surplus areas such as Ali and Naqu in Tibet, and Yushu and Haixi in Qinghai, are mainly located in high-altitude, cold regions with large topographical variations and relatively low human activity intensity, resulting in strong vegetation cover and ecosystem integrity, which significantly enhances soil retention. These areas also have relatively limited tourism development, leading to weak ecological disturbances, further safeguarding their ecological compensation surpluses. In contrast, low-surplus areas in western Inner Mongolia and eastern Xinjiang are affected by dry climates, wind and water erosion, sparse vegetation, and localized tourism development disturbances, resulting in weaker soil retention capacity and limited buffering space for ecosystem disturbances.

The ecological compensation for grassland carbon sequestration and oxygen release by tourism (Fig 9). From 2013 to 2023, in most areas, the ecological compensation for grassland carbon sequestration and oxygen release by tourism remained positive, with the average compensation surplus increasing from 1.42 × 10⁹ CNY to 1.89 × 10⁹ CNY. However, areas with negative compensation were mainly found in individual regions of Inner Mongolia and Xinjiang, with the required average compensation rising from 1.35 × 10⁶ CNY to 1.54 × 10⁷ CNY.

From a spatial distribution perspective, the ecological compensation surplus for carbon sequestration and oxygen release exhibited an “east high, west low” pattern from 2013 to 2023. For instance, in 2023, high-surplus areas were concentrated in Hulunbuir and Xilin Gol League in Inner Mongolia, Guoluo and Yushu in Qinghai, and Naqu, Chamdo, and Shannan in Tibet, while low-surplus areas were primarily found in western Inner Mongolia, northern Qinghai, and southern Xinjiang.

From 2013 to 2023, the ecological compensation for carbon sequestration and oxygen release by tourism was mainly positive, with the average compensation surplus increasing, suggesting that tourism has not created net pressure on grassland carbon sink functions. Instead, it has shown a collaborative enhancing relationship with carbon sequestration and oxygen release functions in grassland ecosystems. However, some regions have seen an increase in ecological compensation deficits, signaling the accumulation of potential ecological risks. The shift from few isolated deficits to multiple regions, particularly in parts of Inner Mongolia and Xinjiang, indicates that in ecologically fragile areas or regions with rapidly expanding tourism, infrastructure development, traffic emissions, and tourist activities have locally overburdened carbon sink functions, reflecting the imbalanced effect of low-carbon industries with high local disturbance [45].

The spatial pattern of high-surplus areas in Hulunbuir, Xilin Gol League, and parts of Qinghai and Tibet, with favorable water-heat conditions or alpine meadow ecosystems supporting strong vegetation cover and biomass, reflects the high carbon sequestration ability. These areas have also adopted relatively eco-friendly tourism development models, which further enhance carbon sink surpluses. Conversely, low-surplus areas in western Inner Mongolia, northern Qinghai, and southern Xinjiang are constrained by arid climates, sparse vegetation, and ecological fragility, limiting carbon sequestration capacity. The overall increase in the number of regions requiring compensation and the shift in ecological compensation amounts indicate that tourism activities and their associated emissions are expanding from the originally fragile western regions to the rapidly developing tourism regions in the east, reflecting the spillover effect of ecological pressure brought on by tourism’s spatial expansion.

## 4. Conclusions and discussion

### 4.1 Research conclusions

(1) From 2013 to 2023, the average supply of direct ecosystem product values in the four major pastoral regions increased from 5.79 × 10¹⁰ CNY to 6.84 × 10¹⁰ CNY, with the spatial pattern showing overall stability in a “high in the southeast and low in the northwest” distribution. Higher and high-level areas were mainly distributed in eastern Inner Mongolia, northwestern Xinjiang, and southwestern Tibet, reflecting that significant improvements in total factor productivity in the livestock sector have enhanced the resource output efficiency per unit of input. Ecological compensation, grazing bans, and grass-livestock balance policies, through a “constraint-incentive” mechanism, have optimized herder behavior, achieving a balance between ecological protection and production efficiency.

(2) Between 2013 and 2023, the average value supply for water conservation remained stable at 9.68 × 10⁶ CNY, while soil retention value decreased from 8.14 × 10⁸ CNY to 8.12 × 10⁸ CNY, carbon sequestration and oxygen release value decreased from 3.84 × 10⁹ CNY to 3.83 × 10⁹ CNY, and air purification value decreased from 1.75 × 10⁵ CNY to 1.72 × 10⁵ CNY. The trends in indirect product value supply across the four pastoral regions indicate that, under the influence of natural constraints, grassland ecosystem regulation functions have remained stable, but their supply capacity has exhibited a marginal weakening trend due to ecological degradation and human disturbance.

(3) Between 2013 and 2023, all regions except Hetian in Xinjiang required ecological compensation for direct grassland products, ranging from 3.59 × 10⁹ CNY to 4.42 × 10⁹ CNY. However, all regions did not need to bear ecological compensation for carbon sequestration and oxygen release by livestock, though the average surplus decreased from 7.38 × 10⁸ CNY to 7.09 × 10⁸ CNY, indicating that direct grassland product supply still strongly supports the realization of value, but the ecological safety margin for carbon sequestration and oxygen release is shrinking.

(4) From 2013 to 2023, most areas in Inner Mongolia and Xinjiang required compensation for the industrial consumption of grassland water conservation, with the average compensation amount decreasing from 2.39 × 10⁷ CNY to 1.23 × 10⁷ CNY. Some areas, such as Alxa League, Hami, and Xining, needed to bear compensation for industrial consumption of grassland carbon sequestration and oxygen release. Most regions in the four pastoral areas had to bear the ecological compensation for air purification by industry, with the average compensation amount decreasing from 2.76 × 10⁷ CNY to 4.44 × 10⁶ CNY. This shows that while the industrial pollution load has been somewhat alleviated, it still exerts continuous pressure on the grassland ecosystem’s regulation functions.

(5) From 2013 to 2023, all regions did not need to bear the ecological compensation for tourism-related grassland water conservation and soil retention. However, areas such as Turpan and Alxa League required compensation for tourism-related carbon sequestration and oxygen release, with the average compensation amount increasing from 1.35 × 10⁶ CNY to 1.54 × 10⁷ CNY, reflecting the increasing localized pressure of tourism activities on carbon sequestration and oxygen release in ecologically fragile areas.

### 4.2 Discussion

(1) Feasibility of Implementing Inter-Industry Ecological Compensation Distribution Mechanisms

Based on the accounting of grassland ecosystem product values, this study constructed an inter-industry ecological compensation distribution model. The ecological compensation amounts calculated for industrial water conservation range from 1.23 × 10⁷ CNY to 2.39 × 10⁷ CNY, and for tourism-related carbon sequestration and oxygen release, from 1.35 × 10⁶ CNY to 1.54 × 10⁷ CNY. In existing studies on ecological compensation amounts across regions, the average ecological compensation varies from 0.2 × 10⁹ CNY to 2.3 × 10⁹ CNY [12,46,47], with the values calculated in this study being slightly lower. This difference arises because the ecological compensation in this study is calculated for a specific industry type’s impact on a single ecosystem service type, and the total value remains within the range of existing studies, ensuring the stability of the inter-industry ecological compensation distribution model to some extent. In terms of the ratio of ecological compensation to industrial output, the average ecological compensation for industrial water conservation constitutes 0.03% of the average industrial output, and for tourism-related carbon sequestration and oxygen release, it constitutes 0.06% of the average tourism output. These results are lower than those obtained by Han Nianlong in his study based on ecosystem service flow-based ecological compensation calculations, where ecological compensation accounted for 0.5% of output value [48]. For the payers of ecological compensation, these amounts represent a minimal economic burden, making them acceptable and thus feasible for implementation.

(2) Spatial Mismatch and Cumulative Pressure of Industrial Impacts on Grassland Indirect Ecosystem Products

This study shows that the industrial impact on grassland ecosystem products in the four pastoral regions exhibits significant functional differentiation and spatial heterogeneity. From 2013 to 2023, the ecological compensation for industrial consumption of water conservation in the southern regions of Qinghai and Tibet remained positive, but the average surplus decreased from 7.36 × 10⁶ CNY to 7.15 × 10⁶ CNY. This indicates that these areas, with strong water conservation capacity and low industrial development intensity, can still cover industrial water pressure, but the reduction in surplus reflects a potential weakening of the ecosystem’s safety margin under increasing industrial loads or climate fluctuations. In contrast, most northern regions, such as Inner Mongolia and Xinjiang, have had negative ecological compensation for water conservation, with compensation amounts decreasing from 2.39 × 10⁷ CNY to 1.23 × 10⁷ CNY. This suggests that industrial water consumption in the arid and semi-arid regions continues to result in net consumption of water conservation, but the pressure has alleviated, possibly due to improvements in water-saving technologies, industrial restructuring, and increased water efficiency. This is consistent with Liu’s findings on water resource pressure in northern China’s arid areas [49].

Meanwhile, the ecological compensation for carbon sequestration and oxygen release showed a generally positive trend, but the average surplus decreased from 1.64 × 10⁹ CNY to 1.29 × 10⁹ CNY, and the number and scale of negative compensation regions increased, indicating that industrial carbon emissions in some ecologically vulnerable areas have exceeded the carbon sink capacity of grasslands. This points to a structural contradiction between energy-intensive industries and ecological vulnerability, as the rate of carbon source expansion outpaces the rate of increase in carbon sinks. This conclusion is commonly found in carbon emission research in China’s northwestern regions [50,51]. Spatially, the surplus for carbon sequestration and oxygen release follows an “east high, west low” pattern, while the negative compensation pressure shows a “west to east shift,” reflecting a significant spatial mismatch between carbon sink capacity and industrial emissions, with high-surplus areas mainly in Hohhot, Xilin Gol League, and parts of Qinghai and Tibet, and low-surplus or deficit areas concentrated in western Inner Mongolia and most of Xinjiang. This is closely related to the differences in vegetation productivity, ecological carrying capacity, and industrial layout [52].

Additionally, the ecological compensation for air purification was predominantly negative, with the average compensation amount decreasing from 2.76 × 10⁷ CNY to 4.44 × 10⁶ CNY, and the size of positive surplus areas continuously shrinking. This indicates that industrial emissions have created long-term and widespread net pressure on grassland air purification services, although this pressure has somewhat alleviated in low-intensity development areas. The spatial pattern shows “east high, west low,” with positive surplus areas extending from “southeast to surrounding areas,” reflecting the mismatch between high pollution intensity regions and the ecosystem’s absorption capacity. It also demonstrates the spillover effect of ecological pressure due to the spatial expansion of industrial activities.

Overall, industrial activities’ impact on grassland ecosystem regulation services in the pastoral regions can be characterized by three main features: (1) spatial mismatch, where high-intensity industrial zones overlap with ecologically vulnerable areas, leading to significant localized ecological pressure; (2) functional dependence, where water conservation, carbon sequestration, and air purification services are constrained by natural conditions and ecological carrying capacity, with limited surplus space and sensitivity to industrial expansion; (3) cumulative pressure, where long-term industrial load accumulates locally, creating ecological deficits that spread to surrounding areas. The study shows that, in the context of shrinking ecological safety margins, relying solely on natural ecosystem self-regulation is insufficient to fully absorb industrial pressures, highlighting the crucial role of human intervention in mitigating ecological risks [53].

To ensure long-term ecological stability in pastoral areas, the following recommendations are made:

Optimize Industrial Layout and Spatial Management: Develop differentiated industrial plans based on ecological carrying capacity and the spatial distribution of water-carbon-air services. Strictly limit the expansion of energy-intensive and high-emission industries into ecologically fragile areas, prioritizing industrial projects in surplus areas to align economic activities with the ecosystem’s service capacity, and alleviate localized ecological pressure.

Enhance Green Production Technologies and Resource Efficiency: Promote water-saving, low-carbon, and clean energy technologies, strengthen industrial emissions control, and improve pollution management to reduce the ecological pressure from industrial activities by improving water efficiency, reducing carbon emissions, and minimizing air pollution.

Establish Dynamic Ecological Compensation and Monitoring Mechanisms: Create a dynamic, cross-regional ecological compensation system to account for the temporal and spatial differences in water conservation, carbon sequestration, and air purification functions. This system should redistribute resources, with high-pressure areas paying and high-surplus areas benefiting. Additionally, continuous monitoring and risk assessment should be used to adjust compensation amounts and management measures in real-time, ensuring sustainable coordination between industrial development and ecosystem services.

## Supporting information

S1 File Dataset. (ZIP)

## References

[1] Wang H, Li Q, Wen Y. The logic and mode of ecological product value realization mechanism: a theoretical analysis based on exclusivity. China Land Sci. 2022;36(04):79–85. doi: 10.11994/zgtdkx.20220214.113646

[2] Ding D, Liang R. Constructing an institutional system for realizing the value of grassland ecological products based on externality theory. Acta Prataculturae Sinica. 2023;31(05):1539–45. doi: 10.11733/j.issn.1007-0435.2023.05.029

[3] Yu H, Zhang L, Li D. Practical experiences and insights from domestic and international realizations of ecological product value. Environ Sci Res. 2020;33(03):685–90. doi: 10.13198/j.issn.1001-6929.2019.08.13

[4] Liu Z, Sun J. A comprehensive study on non-governmental actors in shaping grassland ecological compensation within legal frameworks. Sci Rep. 2024;14(1):5489. doi: 10.1038/s41598-024-56146-7 38448478 PMC10917782

[5] Franklin SL Jr, Pindyck RS. Tropical forests, tipping points, and the social cost of deforestation. Ecol Econ. 2018;153:161–71. doi: 10.1016/j.ecolecon.2018.06.003

[6] Zhang B, Feng Q, Lu Z, Li Z, Zhang B, Cheng W. Ecosystem service value and ecological compensation in Qilian Mountain National Park: implications for ecological conservation strategies. Ecol Indic. 2024;167:112661. doi: 10.1016/j.ecolind.2024.112661

[7] Yang Q, Nan Z, Chen Q. Progress in research on grassland ecological compensation in China. Acta Ecologica Sinica. 2020;40(07):2489–95. doi: 10.5846/stxb201901220169

[8] Miao X, Zhao X. Research on ecological compensation based on ecosystem service value on the loess plateau in eastern Gansu province. China Soil Water Conserv. 2023;(05):61–5. doi: 10.14123/j.cnki.swcc.2023.0112

[9] Zhao J, Ge Y, Li Y. Research on the moderate standard of ecological compensation in the Dawen River basin based on ecosystem service value. Arid Zone Res Environ. 2023;37(04):1–8. doi: 10.13448/j.cnki.jalre.2023.081

[10] Li L u, Xia Q iuyue, Dong J ie. Research on horizontal carbon ecological compensation in county-level spaces within the Wuhan urban agglomeration: based on differences in land use carbon budgets. Acta Ecologica Sinica. 2023;43(07):2627–39. doi: 10.5846/stxb202204130976

[11] Zhou Y, Zhou J, Liu H, Xia M. Study on eco-compensation standard for adjacent administrative districts based on the maximum entropy production. J Clean Prod. 2019;221:644–55. doi: 10.1016/j.jclepro.2019.02.239

[12] Deng HK, Zhang XL, Fan WJ. Research on the cross-regional grassland ecological compensation and reward mechanism based on the energy ecological footprint model - taking the pastoral cities in Inner Mongolia Autonomous Region as an example. For Econ. 2022;44(12):5–23. doi: 10.13843/j.cnki.lyjj.20230112.002

[13] Zhang D, Yuan W, Liu P, Ju Z. Vertical and horizontal ecological compensation of the Taihu Lake Basin based on ecosystem services value. Environ Dev Sustain. 2025. doi: 10.1007/s10668-025-06814-z

[14] Sheng J, Xiao Q, Du Z, Zhang Q, liu Y, Xue Y. Research on vertical ecological compensation mechanism based on ecosystem services supply-demand balance—a case study of the Nine Plateau Lakes in Yunnan Province. Environ Dev Sustain. 2026. doi: 10.1007/s10668-026-07464-5

[15] Zhou Z, Sun X, Zhang X, Wang Y. Inter-regional ecological compensation in the Yellow River Basin based on the value of ecosystem services. J Environ Manage. 2022;322:116073. doi: 10.1016/j.jenvman.2022.116073 36049308

[16] He L, Yao L, Sabev Varbanov P. A Bi-level optimization approach to reduce the pollution burden of lake water with ecological compensation. Ecol Indic. 2023;151:110334. doi: 10.1016/j.ecolind.2023.110334

[17] Ren Y, Lu L, Yu H, Zhu D. Game strategies in government-led eco-compensation in the Xin’an River Basin from the perspective of the politics of scale. J Geogr Sci. 2021;31(8):1205–21. doi: 10.1007/s11442-021-1893-1

[18] Yan A, Tian H, Yin Q, Ding X. Dynamic ecological compensation and delayed strategies in China’s coordinated land-sea governance: an evolutionary game analysis. J Sea Res. 2026;209:102660. doi: 10.1016/j.seares.2025.102660

[19] Shen Q, Lu J, Tutore I, Cucari N, Guo Q. How does horizontal ecological compensation promote the coupled development of ecological environment protection and high-quality economy growth? Evidence from China’s circular economy practices. Socioecon Plann Sci. 2025;102:102320. doi: 10.1016/j.seps.2025.102320

[20] Lei G, Zhong C, Zhang J, Zheng Y. Environmental reward-punishment policy and collaborative green innovation in China: lessons for global environmental management. J Environ Manage. 2025;395:127681. doi: 10.1016/j.jenvman.2025.127681 41135401

[21] Zhang W, Bai Y, Yi J, Engel BA, Yin J, Hua E, et al. Establishing a resource allocation compensation framework based on Green Water Credit concept to coordinate up-downstream development. J Clean Prod. 2026;538:147316. doi: 10.1016/j.jclepro.2025.147316

[22] Shan J, Li D, Li J, Hong L. From fighting alone to win‒win cooperation: horizontal ecological compensation in transboundary marine environmental governance. Ocean Coast Manag. 2026;276:108148. doi: 10.1016/j.ocecoaman.2026.108148

[23] Ouyang Z, Zhu C, Yang G, et al. Total ecosystem production accounting: concept, accounting methods, and case studies. Acta Ecologica Sinica. 2013;33(21):6747–61. doi: 10.5846/stxb201310092428

[24] Liu A, Yang Y, Bai Y. Evolution of grassland-livestock balance management model and progress in monitoring technology in China. Chin J Grassl. 2023;45(12):1–10. doi: 10.16742/j.zgcdxb.20230323

[25] Yuan Y, Zhang L, Cui L. Spatial and temporal variation characteristics of water conservation function of ecosystems in the Zoige Plateau. Chin J Ecol. 2020;39(08):2713–23. doi: 10.13292/j.1000-4890.202008.027

[26] Chen Y, Sun Y, Zeng G. Indirect value assessment of grassland ecosystem services in Liaohe nature reserve. Ecol Sci. 2015;34(01):103–9. doi: 10.14108/j.cnki.1008-8873.2015.01.016

[27] Ouyang Z, Wang X, Miao H. Preliminary study on the services and eco-economic values of terrestrial ecosystems in China. Acta Ecologica Sinica. 1999;19(05):19–25. doi: 10.3321/j.issn:1000-0933.1999.05.004

[28] Zhang H, Chen Z, Zhang X, et al. Assessment of vegetation carbon sequestration and influencing factors in the Sanjiangyuan region from 1977 to 2017. J Nankai Univ Nat Sci Ed. 2021;54(04):87–100. doi: 10.12677/sd.2022.126203

[29] Song X, Chang X, Zhao Y. Assessment of carbon sequestration and oxygen production functions and ecological value of orchards in Xi’an city. Shaanxi Agric Sci. 2019;65(03):76–9. doi: 10.3969/j.issn.0488-5368.2019.03.020

[30] Sun J, Chu Y, He H. Valuation of ecosystem benefits in Jixi city. North Environ. 2003;(03):31–3. doi: 10.3969/j.issn.1673-1212.2003.03.010

[31] Zhang T, Chen L, Pubu D, et al. Valuation of ecosystem service functions of the Lalu Wetland in Lhasa, Xizang. Acta Ecologica Sinica. 2005;12:3176–80. doi: 10.3321/j.issn:1000-0933.2005.12.009

[32] Zhang J, Huang J, Jiang S, et al. Characteristics of domestic sewage discharge in typical suburban villages and towns in northern China. J Environ Eng. 2024;18(01):13–21. doi: 10.12030/j.cjee.202307065

[33] Drewnik M, Musielok Ł, Prędki R, Stolarczyk M, Szymański W. Degradation and renaturalization of soils affected by tourist activity in the Bieszczady Mountains (South East Poland). Land Degrad Dev. 2019;30(6):670–82. doi: 10.1002/ldr.3254

[34] Zhao Y, Pu Y, Lin H, Tang R. Examining soil erosion responses to grassland conversation policy in three-river headwaters, China. Sustainability. 2021;13(5):2702. doi: 10.3390/su13052702

[35] Han Z, Han C, Yang C. Spatial econometric analysis of environmental total factor productivity of ranimal husbandry and its influencing factors in China during 2001-2017. Sci Total Environ. 2020;723:137726. doi: 10.1016/j.scitotenv.2020.137726 32213419

[36] Zhang WN, Ganjurjav H, Liang Y, Gao QZ, Wan YF, Li Y, et al. Effect of a grazing ban on restoring the degraded alpine meadows of Northern Tibet, China. Rangeland J. 2015;37(1):89–95. doi: 10.1071/rj14092

[37] Zhu Q, Chen H, Peng C, Liu J, Piao S, He J-S, et al. An early warning signal for grassland degradation on the Qinghai-Tibetan Plateau. Nat Commun. 2023;14(1):6406. doi: 10.1038/s41467-023-42099-4 37827999 PMC10570289

[38] Che X, Jiao L, Qin H, Wu J. Impacts of climate and land use/cover change on water yield services in the upper yellow river basin in Maqu County. Sustainability. 2022;14(16):10363. doi: 10.3390/su141610363

[39] Yin Y, Ma D, Wu S. Enlargement of the semi-arid region in China from 1961 to 2010. Clim Dyn. 2018;52(1–2):509–21. doi: 10.1007/s00382-018-4139-x

[40] Scurlock JMO, Hall DO. The global carbon sink: a grassland perspective. Glob Change Biol. 1998;4(2):229–33. doi: 10.1046/j.1365-2486.1998.00151.x

[41] Yu S, Zhang Z. Driving Mechanisms of supply–demand imbalance in key ecosystem services in dust storm‐prone areas: a case study of the hotan oasis. Land Degrad Dev. 2025;37(6):1974–88. doi: 10.1002/ldr.70223

[42] Soussana JF, Allard V, Pilegaard K, Ambus P, Amman C, Campbell C, et al. Full accounting of the greenhouse gas (CO2, N2O, CH4) budget of nine European grassland sites. Agric Ecosyst Environ. 2007;121(1–2):121–34. doi: 10.1016/j.agee.2006.12.022

[43] Deng Y, Ling Z, Jiang W. Urban water bodies in China: spatial distribution patterns, temporal change characteristics, and relationship with economic development. Ecol Indic. 2023;157:111139. doi: 10.1016/j.ecolind.2023.111139

[44] Pang Q, Xu J, Zhou Y, He M. Spatial mismatch and drivers of carbon sequestration services supply-demand in China. Ecol Indic. 2025;173:113389. doi: 10.1016/j.ecolind.2025.113389

[45] Lenzen M, Sun YY, Faturay F, et al. The carbon footprint of global tourism. Nat Clim Change. 2018;8(6):522–8. doi: 10.1038/s41558-018-0141-x

[46] Wu C, Lu R, Zhang P, Dai E. Multilevel ecological compensation policy design based on ecosystem service flow: a case study of carbon sequestration services in the Qinghai-Tibet Plateau. Sci Total Environ. 2024;921:171093. doi: 10.1016/j.scitotenv.2024.171093 38387589

[47] Li Y, Qiao G. Research on the compensation standards for grassland ecology in ecological protection red line areas based on herdsmen’s willingness to pay. Arid Land Res Environ. 2021;35(11):55–60. doi: 10.13448/j.cnki.jalre.2021.297

[48] Han NL, Li NH, Li XQ. Research on ecological compensation accounting based on ecosystem service flow: a case study of 18 cities and counties in Hainan Island. J Ecol Rural Environ. 2025, 41(02): 205–14. doi: 10.19741/j.issn.1673-4831.2024.0063

[49] Liu Y, Du J, Ding B, Liu Y, Liu W, Xia A, et al. Water resource conservation promotes synergy between economy and environment in China’s northern drylands. Front Environ Sci Eng. 2021;16(3). doi: 10.1007/s11783-021-1462-y

[50] Wang Y, Zhang L, Zhang Y, Zhong W, Pei K, Qiao W, et al. Evolution characteristics of rural carbon emissions in Northwest China from 2006 to 2019. Environ Res Commun. 2023;5(10):105002. doi: 10.1088/2515-7620/acfd8a

[51] Han F, Kasimu A, Wei B, Zhang X, Aizizi Y, Chen J. Spatial and temporal patterns and risk assessment of carbon source and sink balance of land use in watersheds of arid zones in China - a case study of Bosten Lake basin. Ecol Indic. 2023;157:111308. doi: 10.1016/j.ecolind.2023.111308

[52] Jia C, Yan P, Liu P, Li Z. Energy industrial water withdrawal under different energy development scenarios: a multi-regional approach and a case study of China. Renew Sustain Energy Rev. 2021;135:110224. doi: 10.1016/j.rser.2020.110224

[53] Xu H, Xu F, Lin T, Xu Q, Yu P, Wang C, et al. A systematic review and comprehensive analysis on ecological restoration of mining areas in the arid region of China: challenge, capability and reconsideration. Ecol Indic. 2023;154:110630. doi: 10.1016/j.ecolind.2023.110630

